# CUSP06, a Novel CDH6-Targeted Antibody-Drug Conjugate, Demonstrates Antitumor Efficacy in Multiple CDH6-Expressing Human Cancer Models

**DOI:** 10.3390/pharmaceutics17081049

**Published:** 2025-08-13

**Authors:** Wei Lu, Jing Shi, Wentao Zhang, Nicole Covino, Amy Penticoff, Robert Phillips, John Cogswell, Laurie Tatalick, Stephanie Pasas-Farmer, Jianjian Zhang, Caiwei Chen, Yixuan Wang, Huiyan Shi, Shuhui Liu, Xun Meng, Eric Slosberg

**Affiliations:** 1 OnCusp Therapeutics, 433 Broadway, New York, NY 10013, USA; wentao.zhang@oncusptx.com (W.Z.); nicole.covino@oncusptx.com (N.C.); amy.penticoff@oncusptx.com (A.P.); robert.phillips@oncusptx.com (R.P.); john.p.cogswell@gmail.com (J.C.); laurie@ltatalickconsulting.com (L.T.); spfarmer@bdatasolutions.com (S.P.-F.); 2 Multitude Therapeutics, Building No. 10, 159 Tianzhou Road, Xuhui District, Shanghai 200235, China; jing.shi@multitudetherapeutics.com (J.S.); jianjian.zhang@multitudetherapeutics.com (J.Z.); caiwei.chen@multitudetherapeutics.com (C.C.); yixuan.wang@multitudetherapeutics.com (Y.W.); huiyan.shi@multitudetherapeutics.com (H.S.); xun.meng@multitudetherapeutics.com (X.M.); 3 Multitude Therapeutics, 3698A Haven Ave, Redwood City, CA 94063, USA; shuhui.liu@multitudetherapeutics.com

**Keywords:** CDH6, cadherin-6, K-cadherin, ADC (antibody-drug conjugate), ovarian cancer, exatecan, cholangiocarcinoma, uterine cancer

## Abstract

**Background/Objectives:** Cadherin-6 (CDH6), also known as K-cadherin, is a type II classic cadherin molecule that plays an important role in the embryonic development of the kidney but has very limited expression in adult tissues. It is overexpressed in several human malignancies, primarily in ovarian cancer, renal cell carcinoma, as well as, less frequently, cholangiocarcinoma, uterine serous carcinoma, glioma, lung, pancreatic and thyroid cancers. The characteristic of limited expression in normal tissues, high expression in tumor tissues, and rapid internalization upon antibody binding makes CDH6 a well-suited antibody-drug conjugate (ADC) target. **Methods:** We developed a novel CDH6-targeting ADC, CUSP06, consisting of a proprietary humanized antibody selective for CDH6, a protease cleavable linker, and an exatecan payload, with a drug-to-antibody ratio (DAR) of 8. We further characterized the pharmacological activities of CUSP06 in multiple in vitro and in vivo models. **Results:** CUSP06 was selectively bound to cell surface CDH6 and was efficiently internalized into CDH6-positive ovarian cancer cells, and led to the induction of DNA damage and apoptosis of CDH6-positive cancer cells. CUSP06 exhibited strong antiproliferative activity against several CDH6-positive cancer cell lines and demonstrated strong bystander cell killing effect in the cell mixing experiments in vitro. CUSP06 exhibits excellent in vivo antitumor efficacy in CDH6-high or -low cell line-derived xenograft (CDX) or patient-derived xenograft (PDX) models from human ovarian, renal and uterine cancers, as well as cholangiocarcinoma. CUSP06 demonstrated a favorable safety profile in GLP-compliant toxicology studies in Sprague Dawley rats and cynomolgus monkeys. **Conclusions:** The preclinical data highlighted the therapeutic potential of CUSP06 in multiple CDH6-positive human cancers.

## 1. Introduction

Antibody-drug conjugates (ADCs) represent a promising cancer treatment modality that enables the selective delivery of therapeutic payloads to tumors. Since the first FDA-approved ADC, Mylotarg, for the treatment of CD33-positive AML in 2000, there have been eleven FDA-approved ADCs and over 200 ADCs currently being developed in clinical trials [1,2]. The classic ADC is a tripartite molecule consisting of a humanized monoclonal antibody (mAb), a cytotoxic payload, and a linker that covalently tethers the antibody and payload. The mAb portion of the ADC recognizes a specific antigen on the surface of cancer cells and facilitates the internalization of the ADC into cells. Linkers are divided into two categories: non-cleavable and cleavable [3]. Non-cleavable linker-based ADCs are internalized, and the antibody portion is degraded by lysosomal protease to release the active molecule. Cleavable linkers can be further subdivided into non-enzymatic linkers and enzyme-cleavable linkers. The non-enzymatic cleavable linkers include disulfide-containing linkers that react with thiols to release the active payload, and hydrazone linkers cleaved in an acidic environment to release the active payload. Enzyme-cleavable linkers are processed by lysosomal enzymes, which leads to the release of the cytotoxic payload into the cytoplasm. The cytotoxic payload ultimately results in the killing of cancer cells depending on the mechanistic nature of the payload (e.g., microtubule-disrupting or DNA-damaging). The topoisomerase I inhibitors (e.g., exatecan derivative or DXd) have shown promise in ADC development since the approval of trastuzumab deruxtecan (T-DXd) not only in HER2-high, but HER2-medium or -low human cancers [4]. The TOPO1 payload also possesses desirable “bystander” cell killing effect, in which the released payload is capable of penetrating cell membranes by passive diffusion and impacting the surrounding cancer cells regardless of the antigen target expression level [5,6]. The bystander effect is believed to be an important feature for the ADCs to treat human solid cancers where antigen expression is highly heterogenous. Therefore, many TOPO1 payload-containing ADCs may be therapeutically useful in both high- and low-antigen expression settings.

CDH6, also known as K-cadherin, is a type II classic cadherin molecule that plays an important role in the embryonic development of the kidney but has limited expression in adult tissues. In healthy adults, CDH6 is expressed at low levels in kidney, mammary gland, and thymus, while higher expression is seen in a majority of patients with recurrent ovarian cancer [7,8,9] and renal cell carcinoma [10], as well as, less frequently, across other solid tumors including cholangiocarcinoma, uterine serous carcinoma, glioma, lung, pancreatic and thyroid cancers [11,12,13]. High expression of CDH6 is reported to correlate with tumor progression and poor prognosis in osteosarcoma, low-grade glioma and glioblastoma multiforme [13,14]. CDH6 undergoes rapid internalization upon antibody binding and its membrane expression is restored upon washout of CDH6 antibody [8]. The characteristics of limited expression in normal tissues, high expression in tumor tissues, and rapid internalization upon antibody binding makes CDH6 an ideal ADC target. Two CDH6-targeted ADCs have entered into human clinical trials. HKT288, a CDH6-targeted ADC containing a DM4 payload, was terminated due to unexpected neurologic toxicities [15]. Raludotatug deruxtecan (R-DXd), a CDH6-targeted ADC with the topoisomerase 1 inhibitor DXd, is currently in clinical trials for ovarian, renal and endometrial cancers, and exhibits promising clinical activity in ovarian cancer patients [16].

We report here the preclinical data of CUSP06, a CDH6-targeted ADC that consists of the humanized CDH6 monoclonal antibody covalently linked to exatecan via the T1000 linker construct, which contains a valine–alanine linker and a hydrophilic polysarcosine side chain attached to a PABC self-immolative group. CUSP06 has an average drug-to-antibody-ratio (DAR) of 8. CUSP06 selectively interacts with CDH6 and undergoes rapid internalization into cancer cells. Upon internalization, the exatecan is cleaved from CUSP06 by lysosomal enzymes and leads to the induction of DNA damage and apoptosis of CDH6-positive cancer cells. CUSP06 demonstrates CDH6-dependent cell killing activity in a panel of human ovarian cancer lines. CUSP06 also demonstrates strong bystander cell killing effect in an in vitro cell mixing experiment, suggesting it could be an active therapeutic agent in treating heterogenous CDH6-low solid tumors. CUSP06 exhibits excellent in vivo antitumor efficacy ranging from tumor growth inhibition to tumor regression in CDH6-high or -low cell-line-derived xenograft (CDX) or patient-derived xenograft (PDX) models from human ovarian, renal and uterine cancers as well as cholangiocarcinoma. The preclinical data support the further development of CUSP06 in multiple CDH6-expressing human cancers.

## 2. Materials and Methods

### 2.1. Cell Lines

The human cancer cell lines OVCAR3, PA-1, and ES-2 cell lines were purchased from ATCC. The 786-O cell line was purchased from Shanghai Xunqing Biotechnology Co., Ltd. (Shanghai, China). OVCAR3 cells were cultured in RPMI 1640 medium containing 10% fetal bovine serum and 1% Penicillin-Streptomycin. PA-1 cells were cultured in EMEM medium containing 10% fetal bovine serum and 1% Penicillin-Streptomycin. ES-2 cells were cultured in McCoy’s 5a medium containing 10% fetal bovine serum and 1% Penicillin-Streptomycin. 786-O cells were cultured in RPMI 1640 medium containing 10% fetal bovine serum. GFP-expressing ES2 cells (ES2-GFP) was generated by transducing ES-2 cells with GFP-expressing lentivirus. After several rounds puromycin selection, the stable GFP-expressing cell pool was obtained.

### 2.2. Discovery of CUSP06 mAb Against CDH6

Female Balb/c mice (6–8 weeks) were immunized with recombinant human CDH6 protein (AA Thr 19-Ala 615) purchased from ACRO Biosystems (Beijing, China). Blood samples were obtained and the serum titers against CDH6 protein were measured using ELISA. The spleen cells of mice with high serum titers were fused with myeloma cells (SP2/0), and the fused cells were diluted, cultured in 384-well cell culture plates for 10–14 days before CDH6 ELISA screening. The wells with high CDH6 affinity were selected for subcloning to obtain single clone hybridomas. The subclones were further screened by ELISA, and the binding activity for CDH6 was further verified in CDH6-positive OVCAR-3 ovarian cancer cells by flow cytometry. The hybridoma clones with high binding activity were sequenced and the antibody sequences were subsequently humanized by grafting the murine CDRs into human framework regions. The amino acid sequence of CUSP06 mAb was detailed in the patent US20250090677 [17].

### 2.3. Preparation of ADCs

CUSP06 was produced by maleimide conjugation of the T1000-exatecan linker payload to the endogenous cysteines of Fc region of CUSP06 monoclonal antibody. The conjugation and purification manufacturing process was optimized from the published process [18], and includes buffer exchange of the CUSP06 mAb intermediate, reduction in the endogenous cysteines with Tris (2-carboxyethyl) phosphine (TCEP), maleimide conjugation of the mAb intermediate with the linker-payload, then quenching of the conjugation reaction with n-acetyl-L-cysteine (NAC). The ADC is further purified by Ultrafiltration/Diafiltration (UF/DF) and formulated into a histidine buffer solution to a target concentration of 20.0 mg/mL at pH 5.5. CUSP06 mAb Intermediate was manufactured by WuXi Biologics (Shanghai, China), the T1000-exatecan linker payload was manufactured by Shanghai Haoyuan Chemexpress (Shanghai, China) and the CUSP06 ADC Drug Substance Lot X2012P202210001 was manufactured by WuXi XDC (Wuxi, China) at a 200 g scale.

The DAR value of CUSP06 is determined by reversed phase high-performance liquid chromatography (RP-HPLC), with peak identification and assignment by mass spectrometry (MS). Purity is characterized by size exclusion chromatography (SEC).

Additional analytical data is provided in Table 1.

R-DXd biosimilar was produced using the anti-CDH6 antibody (according to the amino acid sequence from patent US20200390900 [19] conjugated with the deruxtecan linker-payload (DC Chemicals, Shanghai, China), DC50025) according to the conjugation procedure published by Daiichi Sankyo. The characterization of R-DXd biosimilar was shown in Supplemental Figure S3. We refer R-DXd biosimilar as R-DXd in the rest of the manuscript.

Isotype control ADCs were prepared using isotype control antibody conjugated with T1000-exatecan linker payload or deruxtecan linker payload. Rituximab (an anti-CD20 antibody) or Bezlotoxumab (an anti-Clostridium difficile toxin B monoclonal antibody) were used as the isotype control antibody. The DAR of Isotype ADC controls are comparable to that of CUSP06 or R-DXd. The purity and drug antibody ratio (DAR) value of two IgG-T1000-e controls are shown in Supplemental Figure S2.

### 2.4. ELISA Assay

The human, monkey, rat and mouse CDH6 recombinant proteins were purchased from Sino Biological and ACRO Biosystems. Human CDH9 and CDH10 recombinant proteins with histidine tags were purchased from Acrobiosystems. For binding assays, flat-bottomed 96-well plates were coated with 100 µL/well of coating solution (1 µg/mL recombinant CDH6, CDH9 or CDH10 in PBS) and incubated overnight at 4 °C. After washing, the plates were blocked with 1% BSA and incubated with CUSP06, CUSP06 mAb, or IgG control for 1.5 h at room temperature. After washing with PBST buffer (PBS + 0.05% Tween 20), an HRP-conjugated anti-human IgG Fc fragment-specific antibody (Abcam (Waltham, MA, USA), ab99759) was added with 1:10,000 dilution and incubated for 1 h. After washing with PBST buffer, SureBlue™ TMB 1-Component Microwell Peroxidase Substrate (KPL) was added, and absorbance at 450 nm was measured using a microplate reader (MD Spectramax M3, San Jose, CA, USA). Data was processed using Graphpad Prism 8 software with a four-parameter fit, to calculate the EC50.

### 2.5. Binding Affinity of CUSP06 to Endogenous CDH6 in OVCAR3 Cells by Fluorescence Activated Cell Sorting (FACS)

OVCAR-3 cells were cultured to reach the cell confluence of 70–80%. Cells were detached by Trypsin and 1 mM EDTA and harvested by centrifugation at 1300 rpm. After washing with PBS, cells were resuspended cells in antibody washing solution and plated in wells at density of 2000 cells per well. Plates were blocked by blocking solution (PBS + 5% BSA and 1% FBS) then incubated 30 min at 4 °C. After blocking, cell pellets were obtained by centrifugation (1300 rpm) and diluted test articles (100 μL per well) were added to the plate following with a 1 h incubation at 4 °C. After 3 cycles of washing with antibody washing solution (EDTA), anti-Human IgG (Fc specific)-FITC Antibody (Sigma, Saint Louis, MO, USA) was added to the plate at 1:200 ratio (100 μL per well) followed with an 45 min dark incubation at 4 °C. After incubation, cells were washed in 3 cycles and resuspended in PBS and loaded into flow cytometer (BD Biosciences, Franklin Lakes, NJ, USA, BD Accuri C6 Plus), the fluorescent signal from stained cells is detected and converted into a digital signal, Data was processed using Graphpad Prism 8 software with a four-parameter fit to calculate the EC50.

### 2.6. Internalization of CUSP06

PA-1 and OVCAR-3 were cultured in the corresponding culture medium to 70–80% confluency. Cells were detached by EDTA and stopped with blocking solution (PBS containing 2% BSA and 1% FBS), the cell pellets were harvested by centrifugation (1300 rpm) and put in an ice box, 9 × 10^6^ of the cells will be resuspend in Eppendorf tube (Corning, Corning, NY, USA)with blocking solution. CUSP06 or IgG ADC control was added to each tube at the final concentration of 50 μg/mL. After incubation at 4 °C for 60 min, the treated cells were then incubated at 37 °C for varying time points (0, 0.5, 1, 2, 4 and 8 h) to initiate the internalization. At the end of each incubation, an equal volume of 8% paraformaldehyde was added then incubate at room temperature for 15 min then transfer the cell pellets to a 96-well U-shaped plate. The cells were resuspended in 200 μL/well PBS in each well. Cells in the plate were stained with Anti-Human IgG (Fc specific)-FITC fluorescent secondary antibody at 1:300 ratio (Sigma, F9512-2ml). The fluorescence intensity was measured with FITC channel via flowcytometry (BD Accuri C6 Plus, BD Biosciences) and the median fluorescence intensity (MFI) was calculated. The internalization rate/efficiency was determined as {(MFI(CUSP06)-MFI (IgG ADC control)} at each time point/{(MFI(CUSP06)-MFI (IgG ADC control))} at 0 h × 100%

### 2.7. In Vitro Cytotoxicity OVCAR3, PA-1 and ES2

OVCAR3, ES-2 and PA-1 were plated at 1000, 750 and 150 cells per well, respectively, in the 384-well cell culture plates with corresponding culturing medium. After the overnight incubation, the diluted test articles were added. Following 6 days of treatment, the cell viability was measured by Cell Counting Kit-8 (Dojindo, Rockville, MD, USA). Data was processed using Graphpad Prism 8 software with a four-parameter fit to calculate the EC50.

### 2.8. In Vitro Bystander Effect (Cell Mixing Experiment)

ES-2-GFP cell pool was generated according to the following process, an ES-2 cells (ATCC) were transduced with GFP-expressing lentivirus. A stable and positive GFP-expressed cell pool obtained through several rounds of selection and screening with puromycin. OVCAR3 cells and ES-2-GFP cells were cultured in a 5% CO2 incubator at 37 °C with corresponding culturing medium (RPMI 1640 + 10%FBS + 1%PS and McCoy’s 5a + 10%FBS + 1%PS), both cells were harvested and diluted in the appropriate cell density (15,000 cells/well for OVCAR-3 and 2000 cells/well for ES-2-GFP) using a complete medium. After overnight incubation, CUSP06, R-DXd and IgG ADC control were diluted to 2.5 nM using the medium containing equal parts of RPMI 1640 and McCoy’s 5a, and 100 μL 2.5 nM, each of the three test articles was added to corresponding well to achieve the final concentration of 1.25 nM. After 5 days incubation, cell pellets were obtained by 0.25% Trypsin-EDTA digestion and centrifugation (2500 rpm) and resuspend in PBS. The amount of GPF-positive expressed cells and GFP-negative cells was determined by flow cytometry (FASC, iQue3, Sartorius, Göttingen, Germany). For flowcytometry cell gating, cell population was first gated by FSC-H and SSC-H then gated by FSC-H and FSC-A, at the end using GFP (BL1-H) and SSC-H to quantify the GFP-positive ES-2 and -negative OVCAR-3 cell populations.

### 2.9. Western Blot: Effect on DNA Damage Pathway (pChk1 and PARP1)

OVCAR-3 cells were cultured in a 5% CO2 incubator at 37 °C using complete medium (RPMI 1640 + 10%FBS + 1% Penicillin-Streptomycin). Cells were harvested by using 0.25%Trypsin-EDTA and centrifugation (1000 rpm for 5 min) when confluency reached 70%, The cells were then resuspended in medium and plated at a density of 4 × 10^5^ cells/well with a volume of 2 mL per well. Exatecan was diluted with DMSO to a concentration of 5 µM, and CUSP06 mAb, IgG-ADC control, and CUSP06 ADC were diluted with complete medium to a concentration of 1 µM, the diluted test articles were added with 2 µL per well. After incubating at 37 °C with 5% CO_2_ for 3 days, cells were lysed in RIPA buffer containing protease and phosphatase inhibitors (Boston BioProducts, Milford, MA, USA). After centrifuging the lysates at 12,000 rpm for 10 min at 4 °C, the protein concentration was measured using the BCA Protein Assay Kit (SolarBio, Beijing, China). The supernatant was combined with 4× loading dye and 10× reducing agent to each sample and denatured at 95 °C for 10 min, 30 µg protein per well was then loaded on a NuPAGE™ 4–12% Bis-Tris Midi Gel (Invitrogen, Carlsbad, CA, USA). After 2 h electrophoresis at 125 V, the proteins were transferred to a nitrocellulose membrane via Blot^®^2 (Invitrogen). Western blot analysis was conducted with the following primary antibodies: Phospho-Chk1 (Cell Signaling Technology, Danvers, MA, USA, (CST) 2348), total Chk1 (CST, 2360), Phospho-H2AX (CST 9718), cleaved PARP (CST 5628), and β-actin (CST 3700). The secondary antibody (IR-Dye 800 CW Goat anti Rabbit IgG (H + L), LICOR) was added to the membrane and incubated at RT for 10 min. The membrane was scanned using LI-COR Odyssey and analyzed with Image Studio Lite Ver 5.2.

### 2.10. In Vitro Stability in Plasma

The plasma samples from rats, mice, monkeys, and humans were obtained at Center for Drug Safety Evaluation and Research, Shanghai Institute of Materia Medica. The plasma stability testing samples were prepared under sterile conditions by spiking CUSP06 into a pooled plasma (K2EDTA) of each species as well as in ELISA dilution buffer (1% BSA-PBST) at the nominal concentrations of 1 mg/mL. Samples were aliquoted and stored at 37 °C for 0, 1, 3, 5, 7, 10, 14 and 21 days. At the designated time point, stability samples were frozen and stored at −65 °C or lower. All samples were analyzed in duplicate by HPLC-MS/MS methods for exatecan at the end of the study. The concentrations of exatecan in plasma were determined by the HPLC-MS/MS methods.

### 2.11. CDX Model Studies

The 6–8 weeks old female BALB/c mice were purchased from Shanghai Lingchang Biotechnology Co., Ltd. (Shanghai, China). and Laboratory Animal Management Department of Shanghai Family Planning Research Institute for OVCAR3 and PA-1 CDX studies, respectively, The 6–8 weeks old female mice NCG (NOD/ShiLtJGpt-Prkdcem26Cd52Il2rgem26Cd22/Gpt) were purchased from Jiangsu GemPharmatech Co., Ltd. (Najing, China). for 786-O CDX study. CDX models were created by injecting cell suspensions (OVCAR3, PA-1 or 786-O) into female mice subcutaneously, either in Matrigel or saline. Animals were randomized into treatment and control groups (N = 5 per group), with dosing initiated on day 0 when tumor volumes reached approximately 100–250 mm^3^, Tumor volume and body weight was measured twice a week. Vehicle control groups received PBS buffers. All procedures were approved by WuXi AppTec and Multitude Therapeutic’s Institutional Animal Care and Use Committee.

### 2.12. PDX Model Studies

In vivo efficacy studies with ovarian cancer, kidney nephroblastoma, and cholangiocarcinoma patient-derived xenograft (PDX) models were conducted at Lide Biotech Co. (Shanghai, China), Ltd. For patient-derived xenograft (PDX) studies, female 6–8 weeks old nude mice (Nu/Nu) were purchased from Beijing Vital River Laboratory Animal Technology Co., Ltd. (Beijing, China) and Zhejiang Charles River Co., Ltd. (Jiaxing, China). Models were established by subcutaneously inoculating of tumor tissue fragments obtained from patients and maintained in host mice. Animals were randomized into treatment and control groups, with dosing initiated on day 0 when tumor volumes reached approximately 100–250 mm^3^. Tumor volume and mouse body weight were measured twice a week. Vehicle control groups received PBS buffers. All procedures were approved by Lide Biotech Co., Ltd. (Shanghai, China) Institutional Animal Care and Use Committee.

The in vivo efficacy study with uterine PDX model was conducted in Crown Bioscience (Taicang) Inc. (Suzhou, China). For patient-derived xenograft (PDX) studies, female 6–9 weeks old Balb/c nude mice were purchased from Jiangsu GemPharmatech Co., Ltd. (Nanjing, China). Model were established by the subcutaneous inoculation of tumor tissue fragments derived from patient sample, which were maintained in host mice. Animals were randomized into treatment and control groups (N = 5 per group), with dosing initiated on day 0 when tumor volumes reached approximately 100–250 mm^3^, Tumor volume and animal body weight were measured was twice a week. Vehicle control groups received PBS buffers. All procedures were approved by Crown Bioscience (Taicang) Inc’s Institutional Animal Care and Use Committee.

### 2.13. IHC Analysis of Various Proteins in PDX Tumor Samples

Xenograft tumors were formalin-fixed and paraffin-embedded, and tissue sections were used for Immunohistochemical (IHC) analysis. Antigen retrieval was conducted with EnVision FlEX Target Retrieval Solution (DAKO, Santa Clara, CA, USA). Mouse anti-CDH6 (Ab-Mart, Berkeley Heights, NJ, USA, CL069439), mouse anti-BCRP (Abcam ab3380), mouse anti-P-glycoprotein (Abcam ab3366) and mouse IgG isotype control (Abcam ab37355) were used as primary antibodies. Primary antibody was detected with DAKO REAL Envision detection system, Peroxidase/DAB, Rabbit/Mouse HRP kit. The antigen staining intensity of each tumor cell was classified as negative or positive with three levels of intensity (strong, moderate, and weak). Based on the percentage of cells in each staining intensity, and H score is calculated using the following formula: H score = 3 × (percentage of strongly staining cells) + 2 × (percentage of modestly staining cells) + 1 × (percentage of weakly staining cells)

### 2.14. Toxicology Studies in Sprague Dawley Rats and Cynomolgus Monkeys

CUSP06 was intravenously administered once every three weeks with two doses in total, at 0 (saline control) 60, 100, and 150 mg/kg in Sprague Dawley rats (15 animals/gender/group), or at 0 (saline control), 10, 20, and 30 mg/kg in cynomolgus monkeys (five animals/gender/group). Two thirds of animals/gender/group were necropsied at 1-week after the last dose and the remaining one third of animals/gender/group were necropsied at 6-week after last dose. Study endpoints included: morbidity and mortality, clinical observations, ophthalmology, body weight, food consumption, hematology, coagulation, plasma chemistry, urinalysis, local irritation at administration site, neurologic system safety pharmacology, sample collection for bioanalysis (ADC, Total antibody [TAb: unconjugated and conjugated antibody] and free unconjugated payload [exatecan]) and toxicokinetic evaluations, immunogenicity assessment (anti-drug antibody [ADA]), gross pathology, organ weights and histopathology.

### 2.15. Bioanalytical Method of CUSP06 ADC, Total Antibody and Exatecan Free Payload in Monkey and Rat

The plasma concentration of CUSP06 total antibody was measured by ELISA where total antibody was captured by His-tagged recombinant CDH6 (Acro Biosystems, Beijing, China) followed by anti-human IgG-heavy and light chain monkey-absorbed-HRP conjugated detection antibody (Bethyl Laboratory, Montgomery, AL, USA). The HRP substrate, Tetramethylbenzidine (TMB) was added and the color intensity was measured at 450 nm. The LLOQ is 0.156 µg/mL

The plasma concentration of CUSP06 ADC was measured by ELISA where the ADC was captured by Anti-exatecan antibody (Abmart, Berkeley Heights, NJ, USA) followed by the mixture of His-tagged recombinant CUSP06 protein (Acro Biosystems) and HRP labeled-anti-His-antibody (GenScript, Nanjing, China). The HRP substrate, Tetramethylbenzidine (TMB) was added, and the color intensity was measured at 450 nm. Analyte concentrations were calculated from the standard curve. The LLOQ is 1.25 µg/mL.

Exatecan free payload was extracted from plasma sample using ethyl acetate. Exatecan-d5 was used as internal standard. After evaporation to remove the organic solvent, the residue was dissolved with 1% Formic acid in methanol/water (1:1) and analyzed with a Shimadzu (Columbia, MD, USA) LC-30AD HPLC and an AB SCIEX (Marlborough, MA, USA) Triple Quad 6500 + Mass Spectrophotometer. Data was acquired and integrated with Analyst 1.7 and was quantitated with Watson LIMS 7.5 SP1. The LLOQ was 50 pg/mL.

## 3. Results

### 3.1. CUSP06 Is a Novel and Selective CDH6-Targeted ADC

**Figure 1:**
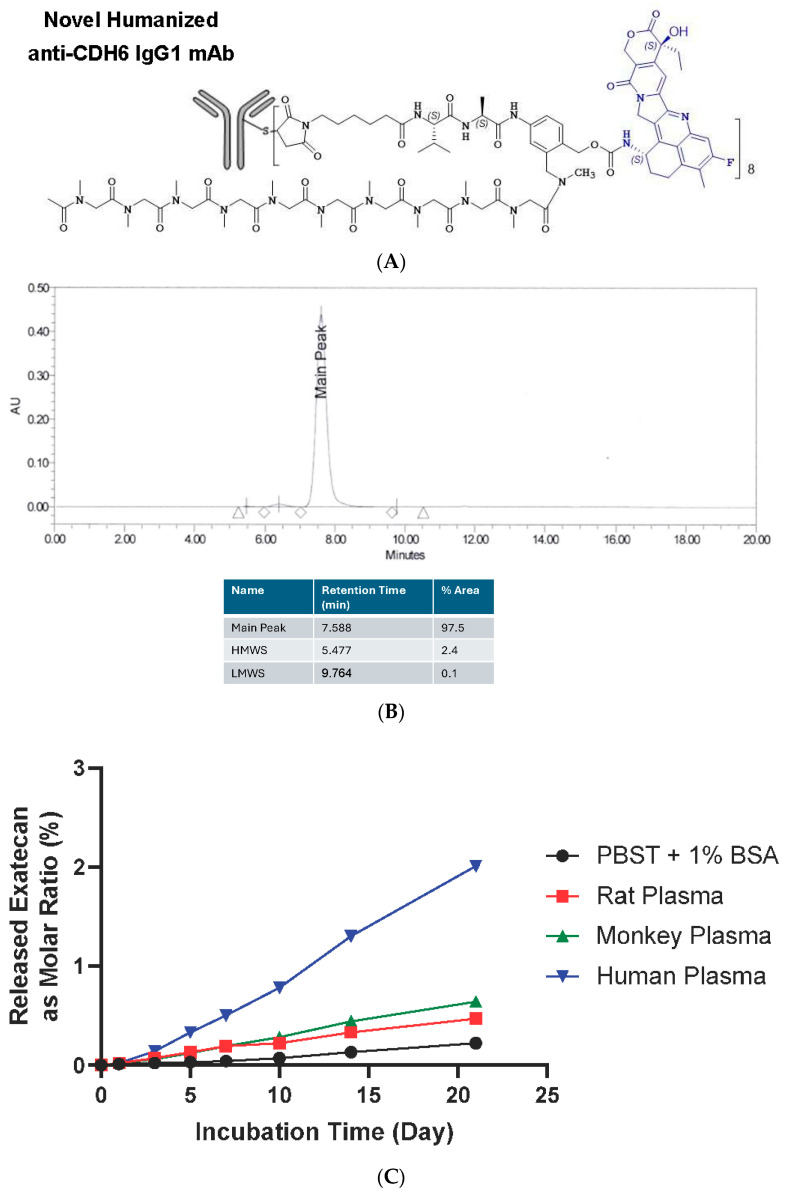

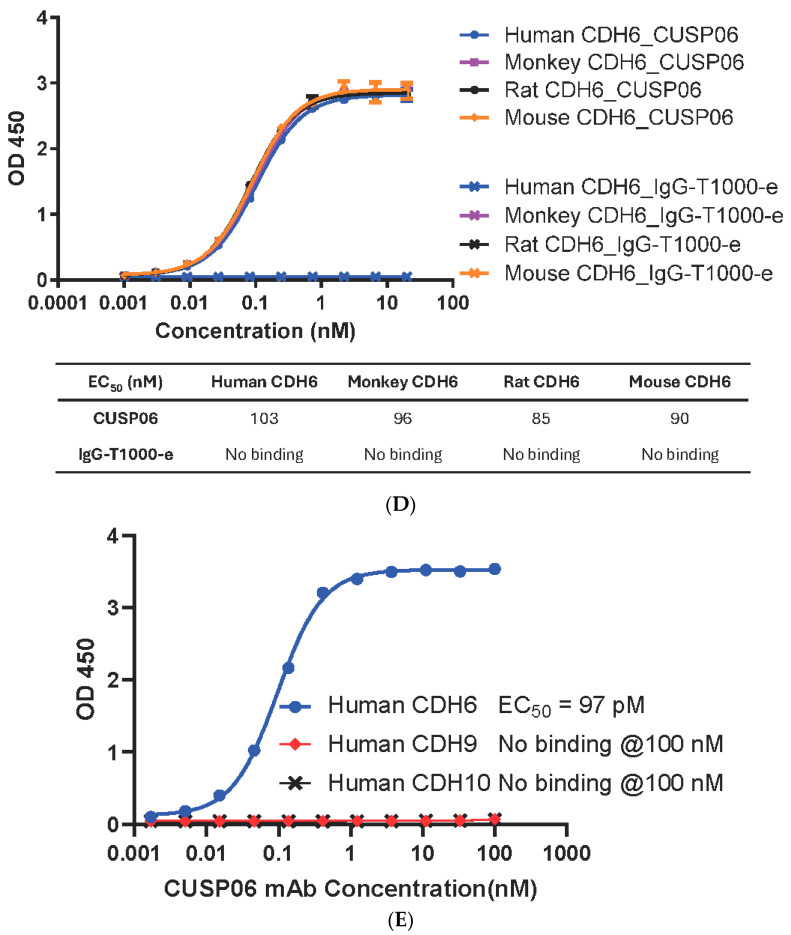
Structure, property and selectivity of CUSP06. (**A**) Schematic structure of CUSP06. (**B**) Size exclusion chromatogram of CUSP06 indicates CUSP06 is homogenous in solution. (**C**) In vitro plasma stability of CUSP06 in buffer (PBST + 1% BSA), human, rat and monkey plasma. (**D**) Binding affinity of CUSP06 to human, monkey, rat and mouse CDH6. (**E**) Binding selectivity of CUSP06 for human CDH6, CDH9 and CDH10 by ELISA.

A novel anti-human CDH6 mAb was generated by immunizing Balb/c mice with a recombinant human CDH6 protein. In order to minimize immunogenicity in humans, the murine CDRs were subsequently grafted onto human IgG1_K_ to yield humanized CDH6-targeting monoclonal antibody (mAb). CUSP06 is composed of three components: (1) the humanized CDH6-targeting IgG1_K_ monoclonal antibody (CUSP06 mAb) covalently linked to, (2) a topoisomerase inhibitor payload (exatecan), via, (3) the T1000 linker construct containing a protease-cleavable valine–alanine linker and a polysarcosine modified para-aminobenzyl carbamate (PABC) self-immolative spacer (Figure 1A). The average drug-to-antibody ratio (DAR) of 8 was selected for CUSP06 given the restricted CDH6 expression in normal tissues. CUSP06 is a homogenous ADC based on size exclusion chromatography (Figure 1B), with all eight endogenous cysteines conjugated to the linker payload, yielding a fully conjugated IgG1 antibody with a DAR of 7.9 as characterized by RP-HPLC-MS (Supplemental Figure S1). The T1000-exatecan linker payload has been shown to be stable [18]. As expected, the molar percentage of exatecan payload released from CUSP06 was low, with only 0.6, 0.9 and 2.0% in rat, monkey and human plasma over 21 days (Figure 1C).

The CUSP06 mAb showed similar affinity to recombinant CDH6 protein from human, monkey, rat and mouse by ELISA, with EC50s of 64 ± 7 pM (N = 3), 61 ± 5 pM (N = 3), 57 ± 6 pM (N = 3), and 63 ± 4 pM (N = 3), respectively. CUSP06 exhibited comparable affinity to CDH6 from human, monkey, rat and mouse, with EC50s of 95 ± 3 pM (N = 3), 88 ± 3 pM (N = 3), 81 ± 5 pM (N = 3), and 85 ± 2 pM (N = 3), which indicated the conjugation of T1000-exatecan linker-payload did not interfere with the CDH6 binding (Figure 1D). The similar binding affinity of CUSP06 to CDH6 proteins from human, monkey and rat supports the selection of monkey and rat as the preclinical species for the nonclinical toxicology studies.

The binding affinity of CUSP06 mAb and CUSP06 to endogenous human CDH6 was confirmed by a Fluorescence Activated Cell Sorting (FACS) method using CDH6-expressing human ovarian cancer cell line OVCAR-3. CUSP06 mAb bound endogenous CDH6 with EC_50_ of 280 ± 136 pM (N = 3). CUSP06 showed similar binding affinity to CDH6 with EC_50_ of 406 and 683 pM (average EC_50_ = 545 pM).

CDH9 and CDH10 are the two most closely related cadherins in the human proteome with 74% amino acid sequence homology to the extracellular domain of CDH6. In a head-to-head comparison experiment, CUSP06 mAb showed high binding affinity with human CDH6 with EC50 of 97 pM. In contrast, it showed no binding affinity to CDH9 or CDH10 even at 100 nM, the highest concentration tested (Figure 1E). These data support that CUSP06 mAb is a highly selective CDH6 antibody.

### 3.2. CUSP06 In Vitro Pharmacology

**Figure 2:**
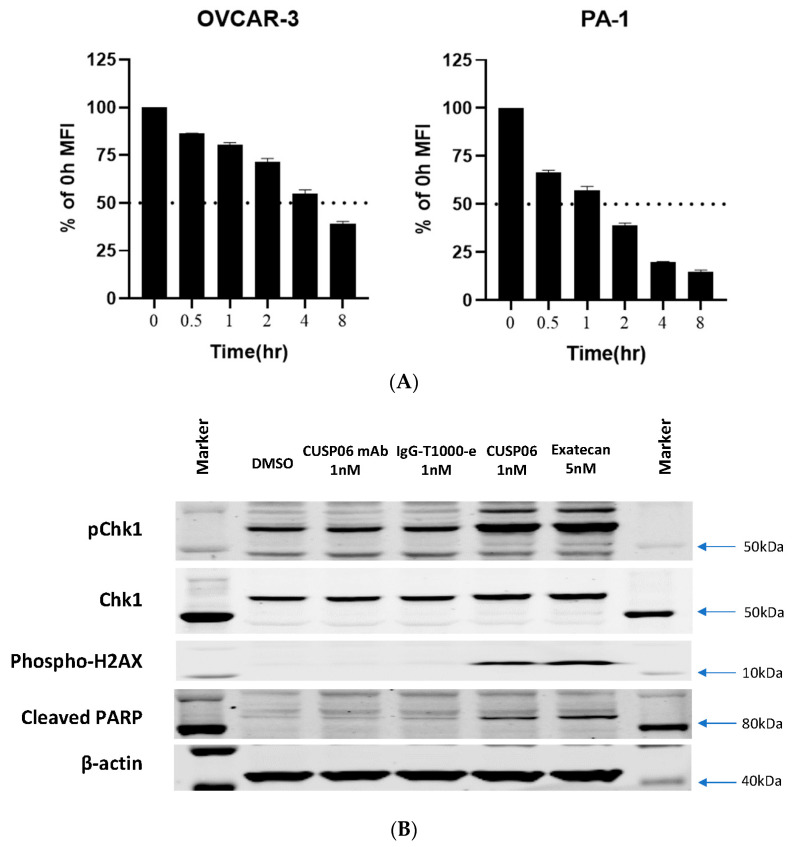

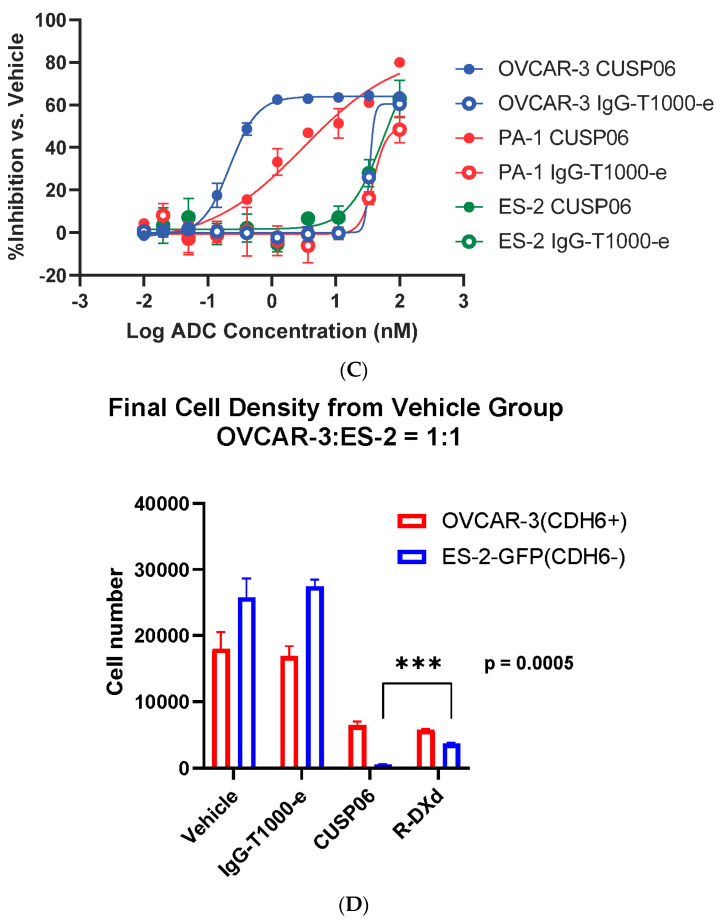
In vitro Characterization of CUSP06. (**A**) Internalization of CUSP06 in two ovarian cancer cell lines, OVCAR3 and PA-1. (**B**) Determination of DNA damage and apoptosis caused by CUSP06 in OVCAR3 cells. After OVCAR-3 cells were treated with CUSP06, CUSP06 mAb, IgG-ADC control or exatecan for 72 h, pChk1, total Chk1, p-H2AX, cleaved PARP, and β-actin level were detected by Western Blot. (**C**) Characterization of the antiproliferative activities of CUSP06 in CDH6-positive (OVCAR3 and PA-1) and CDH6-null ES2 cells by CCK8 assay. (**D**) Characterization of in vitro bystander effect of CUSP06. A mixture of OVCAR3 cells and ES-2-GFP cells were treated with 1.25 nM IgG-ADC, CUSP06, or R-DXd for 5 days. The viable cell numbers were determined by FACS analysis.

Rapid internalization of an ADC is critical for its antiproliferative activity in vitro and in vivo. CUSP06 exhibited fast internalization kinetics in the two CDH6-expression ovarian cancer cell lines, PA-1 and OVCAR3, achieving 50% internalization between 1 and 4 h of treatment (Figure 2A).

The topoisomerase inhibitor exatecan has previously been shown to induce double--strand DNA breaks and causes apoptosis in cancer cells [20]. Incubation of CDH6-expressing OVCAR3 cells with CUSP06 orexatecan for 72 h resulted in increased level of DNA damage biomarkers (phospho-Chk1 and phospho-H2AX) and a marker of apoptosis (cleaved PARP) (Figure 2B). In contrast, the CUSP06 mAb alone or Isotype ADC control did not cause any DNA damage or apoptosis effects. The data indicated that the induction of DNA damage and apoptosis by exatecan released from CUSP06 is one of the cytotoxic mechanisms of CUSP06 against CDH6-expressing cancer cells (Figure 2B).

The in vitro antiproliferative activity of CUSP06 was evaluated in two CDH6-expressing human ovarian cancer cell lines, OVCAR-3 and PA-1, and one CDH6 null ovarian cancer line ES-2. CUSP06 showed potent antiproliferative activity in the two CDH6-expressing cancer cell lines, with an average GI50 of 0.45 nM (N = 4) and 5.1 nM (N = 4) to OVCAR-3 and PA-1 cells, respectively. IgG-T1000-e ADC control showed much reduced activity with GI50 > 50 nM in OVCAR3 and PA1 cell lines. CUSP06 exhibited similar antiproliferative activity in ES-2 cells compared to IgG-T1000-e ADC control (GI50 > 50 nM), which was likely caused by nonspecific uptake of the ADC molecule (Figure 2C). The in vitro antiproliferation data showed the CDH6-dependent cell killing activity by CUSP06 in ovarian cancer cell lines.

One of the drivers of the excellent clinical activity of Enhertu (trastuzumab deruxtecan) in HER2-low breast cancer patients is the potent bystander effect of the DXd payload [21]. To assess the bystander effect of CUSP06, a cell mixing experiment was conducted by incubating a mixture of CDH6-expressing OVCAR3 cells and GFP-labeled CDH6-null ES-2 cells with CUSP06 for 5 days. Like the parent ES-2 cells, the GFP-labeled ES-2 cells were insensitive to the treatment of CUSP06. CUSP06 treatment resulted in not only the decreased number of viable OVCAR3 cells, but also the reduced number of viable ES-2 cells. The result supported that CUSP06 was taken up by CDH6-positive OVCAR3 cells, resulting in cell death. The free payload cleaved within and released from dead OVCAR3 cells then permeated into CDH6-negative ES-2 cells, subsequently killing them (Figure 2D), indicating a strong bystander effect of CUSP06. Since R-DXd is reported to show a good bystander effect [8], a side-by-side comparison of CUSP06 and R-DXd confirm not only the bystander effect of R-DXd, but also shows that the bystander effect of CUSP06 is significantly stronger. This is consistent with the reports that exatecan possesses better cell permeability than DXd [21], and that MTX-1000 (Trastuzumab-T1000-e) showed stronger bystander effect than trastuzumab deruxtecan [18]. The data indicate that CUSP06 could have strong antitumor efficacy in CDH6-low tumors.

### 3.3. Antitumor Activity of CUSP06 in Ovarian and Renal Cancer CDX Models

**Figure 3:**
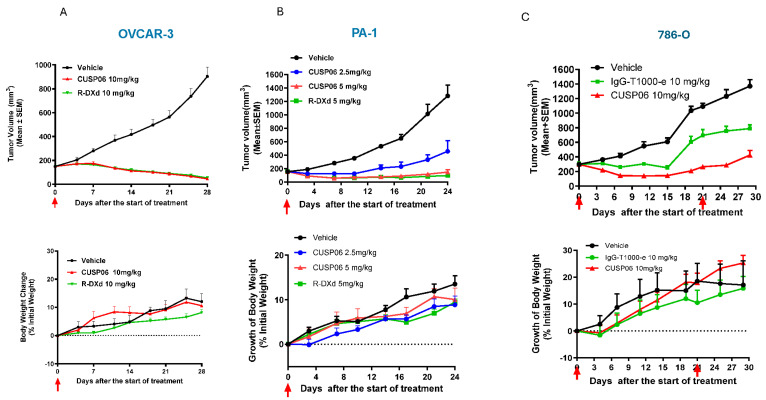
In vivo antitumor activity of CUSP06 in CDX models. (**A**) Antitumor activity of CUSP06 in OVCAR3 xenograft model. A single dose of 10 mg/kg of CUSP06 was administered intravenously to the tumor-bearing mice at Day 0. Each data represents mean and SEM of tumor volume or relative body weight changes. Each group contained six mice. (**B**) Antitumor activity of CUSP06 in PA-1 xenograft model. A single dose of 2.5 or 5 mg/kg CUSP06 was administered intravenously to the tumor-bearing mice at Day 0. Each data represents mean and SEM of tumor volume or relative body weight changes. Each group contained five mice. (**C**) Antitumor activity of CUSP06 in 786-O xenograft model. Two doses of 10 mg/kg of CUSP06 or IgG-T1000-e were administered intravenously to the tumor-bearing mice at Day 0 and D21 of the study. Each data represents mean and SEM of tumor volume or relative body weight changes. Each group contained five mice.

The in vivo antitumor activity of CUSP06 was investigated in three xenograft models in which CDH6 expression level are high: PA-1 (Ovarian Cancer), OVCAR-3 (Ovarian Cancer) and 786-O (Renal Cell Carcinoma). We chose 10 mg/kg dose level for most of the in vivo study since it is an efficacious dose level commonly used for many Topo1 inhibitor payload-containing ADCs [8,18,22]. A single dose of 10 mg/kg CUSP06 significantly regressed tumor growth compared to vehicle control (*p* < 0.001) in the OVCAR-3 CDX model (Figure 3A). A single dose of CUSP06 demonstrated dose-dependent tumor growth regression or inhibition (*p* < 0.001) in the PA-1 CDX model (Figure 3B). CUSP06 demonstrated similar antitumor activity as R-DXd in both ovarian cancer CDX models. CUSP06 also resulted in significant tumor growth inhibition (*p* < 0.01) compared to vehicle or isotype-ADC controls in the 786-O CDX model (Figure 3C). These in vivo pharmacology data indicated CUSP06 is efficacious in multiple CDH6-positive cell line-derived xenograft models. CUSP06 was well tolerated in the in vivo studies as indicated by minimal effect on the animal body weight change compared to vehicle and isotype-ADC control groups. Pharmacokinetic profiling in PA-1 tumor-bearing mice indicated that CUSP06 had a satisfactory pharmacokinetic profile after a single intravenous dose of 5 mg/kg CUSP06 (Table 2). The half-life (T1/2) of the total antibody was 55.0 h, its highest concentration (Cmax) was 115.3 μg/mL, and its AUC0-t was 6714.1 μg/mL*h. The half-life (T1/2) of ADC was 46.2 h, its highest concentration (Cmax) was 112.1 μg/mL, and its AUC0-t was 5649.5 μg/mL*h.

### 3.4. Antitumor Activity of CUSP06 in Ovarian, Renal and Uterine Cancer and Cholangiocarcinoma PDX Models

As patient-derived xenograft (PDX) models grow in a physiologically relevant tumor microenvironment and preserve the heterogeneity of tumor architecture, they have been found to have a better predictive value to patient response to novel therapeutics compared to CDX models [23,24]. Hence, we tested the antitumor activity of CUSP06 in ovarian and renal cancer PDX models. In addition, we evaluated the CUSP06 efficacy in CDH6-positive uterine cancer and cholangiocarcinoma PDX models, where the therapeutic potential of CDH6-targeted ADCs has not previously been demonstrated.

**Figure 4:**
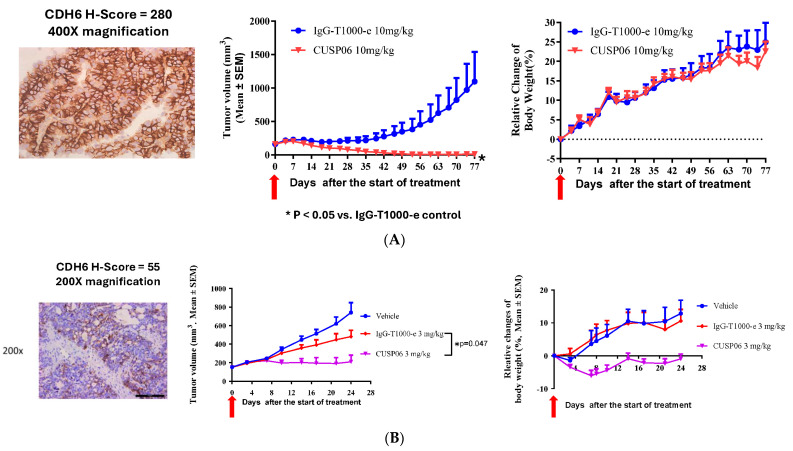

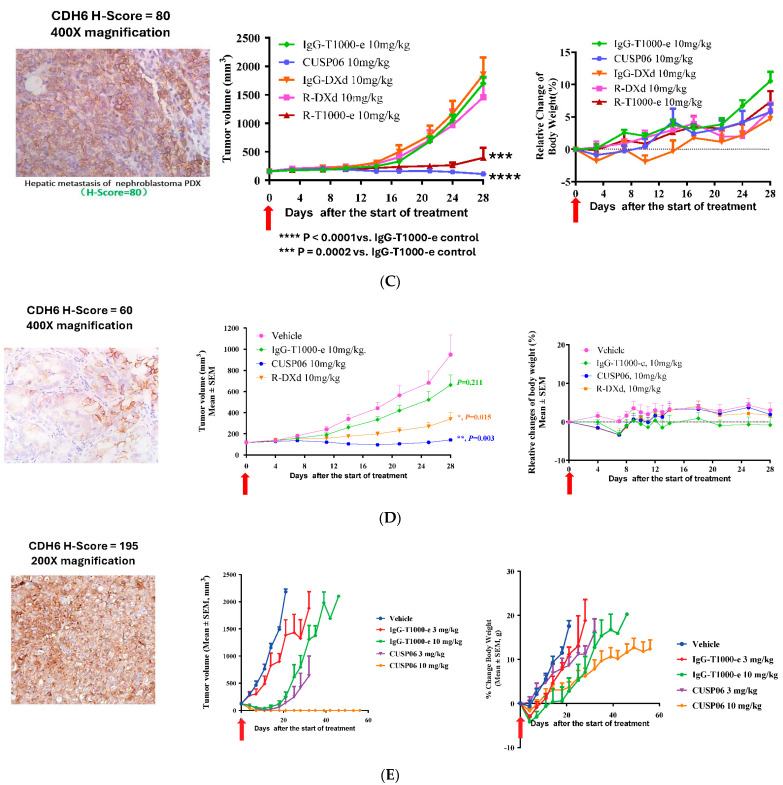
In vivo antitumor activity of CUSP06 in PDX models. (**A**) Antitumor activity of CUSP06 in a CDH6-high Ovarian PDX model (LD-1588). A single dose of 10 mg/mg CUSP06 or IgG-ADC control was administered intravenously to the tumor-bearing mice at Day 0. Each data represents mean and SEM of tumor volume or relative body weight changes. Each group contained five mice. (**B**) Antitumor activity of CUSP06 in a CDH6-low Ovarian PDX model (LD-2851). A single dose of 3 mg/mg CUSP06 or IgG-ADC control was administered intravenously to the tumor-bearing mice at Day 0. Each data represents mean and SEM of tumor volume or relative body weight changes. Each group contained five mice. (**C**) Antitumor activity of CUSP06 in a CDH6-low kidney nephroblastoma PDX model (LD-2511). A single dose of 10 mg/mg CUSP06, R-DXd, R-T1000-e, or IgG-ADC control was administered intravenously to the tumor-bearing mice at Day 0. Each data represents mean and SEM of tumor volume or relative body weight changes. Each group contained five mice. (**D**) Antitumor activity of CUSP06 in a CDH6-low cholangiocarcinoma PDX model (LD-2214). A single dose of 10 mg/mg CUSP06, or IgG-ADC control was administered intravenously to the tumor-bearing mice at Day 0. Each data represents mean and SEM of tumor volume or relative body weight changes. Each group contained five mice. (**E**) Antitumor activity of CUSP06 in a CDH6-medium uterine cancer PDX model (UT3705). A single dose of 3 or 10 mg/mg CUSP06, or IgG-ADC control was administered intravenously to the tumor-bearing mice on Day 0. Each data represents mean and SEM of tumor volume or relative body weight changes. Each group contained five mice.

We identified two ovarian PDX models with different CDH6 levels by immunohistochemistry method. These two models are valuable to investigate the preclinical activity of CUSP06 in CDH6-high and CDH6-low settings. A single dose of 10 mg/kg CUSP06 significantly regressed the tumor growth in a CDH6-high, PARP inhibitor-resistant ovarian PDX model (LD1-1588, CDH6 H-score = 280) compared to isotype-ADC control (*p* < 0.05). Four out of five mice in the CUSP06 treatment group had no measurable tumor at the end of the study (Figure 4A). The PARP inhibitor-resistant ovarian PDX model LD-2851 has lower levels of CDH6 expression with a significant degree of heterogeneity. The CDH6 H-score of 55 consists of 80% of cells are CDH6-negative, 5% of cells have CDH6 2 + staining and 15% of cells have CDH6 3 + staining. CUSP06 also demonstrated significant tumor growth inhibition compared to vehicle or isotype-ADC controls in this model (Figure 4B). CUSP06 was well tolerated in both studies since there was no significant difference in the body weight changes in the CUSP06 treatment group and control groups. These preclinical data support the therapeutic potential for CUSP06 in CDH6-low and CDH6-high ovarian cancer.

In a CDH6-low kidney nephroblastoma PDX model (LD-2511, CDH6 H-score = 80) a single dose of 10 mg/kg CUSP06 significantly repressed the tumor growth compared to the IgG-T1000-e control (Figure 4C). The H-score of 80 consists of 40% of cells that are CDH6-negative, 40% of cells that have CDH6 + 1 staining and 20% of cells that have CDH6 + 2 staining. The data indicated that CUSP06 of capable of killing not only the CDH6-expressing tumor cells, but also the CDH6-negative tumors cells likely via bystander killing effect and proximity near the CDH6-expressing cells in this heterogeneous CDH6-expressing PDX model. Interestingly, a single dose of 10 mg/kg R-DXd failed to show appreciable antitumor activity compared to the corresponding Isotype ADC control (IgG-DXd). Replacement of the DXd linker payload with T1000-e partially restores the antitumor activity in this model compared to IgG-T1000e control. DXd is a known substrate for efflux pumps such as P-gp and BCRP [25]. Since this PDX model lacks P-gp or BCRP expression by IHC analysis (Supplemental Figure S4), the difference in the antitumor activity between CUSP06 and R-DXd is likely due to the fact that the CUSP06 payload, exatecan, is a more potent toxin with higher cell permeability and improved bystander effect compared to DXd.

A single dose of 10 mg/kg CUSP06 significantly inhibited tumor growth in a CDH6-low cholangiocarcinoma PDX model (LD-2214, CDH6 H-score = 60) compared to the vehicle or IgG-T1000-e control groups (*p* < 0.005) (Figure 4D). In addition, CUSP06 demonstrated stronger in vivo antitumor activity than R-DXd (*p* < 0.01). Therefore, CUSP06 has the potential to be more efficacious than R-DXd in CDH6-low cancers. CUSP06 also demonstrated dose-dependent tumor growth inhibition in a CDH6-medium uterine cancer PDX model (UT-3705, CDH6 H-score = 195). A single dose of 10 mg/kg CUSP06 resulted in absence of any measurable tumor in all five host mice up to day 56 post dose (Figure 4E). This is the first time to our knowledge that a CDH6-targeted ADC has demonstrated antitumor efficacy in settings other than ovarian cancer and RCC and indicate CUSP06 could have therapeutic potential in CDH6-positive uterine cancer and cholangiocarcinoma patients.

### 3.5. CUSP06 Showed an Acceptable Safety Profile in GLP Toxicology Studies with Sprague Dawley Rats and Cynomolgus Monkeys

Rats and monkeys were identified as relevant species for toxicity testing based on similar binding affinity of CUSP06 to CDH6 from rats, monkeys and humans.

CUSP06 was well tolerated at all doses tested in SD rats. No CUSP06-related local irritation findings were found at any dose level, and no abnormal effects on central nervous system were noted. Target organs included lymphoid and hematopoietic organs (bone marrow, thymus, spleen, and mesenteric and inguinal lymph nodes) and reproductive system (testes, epididymides and mammary gland in males, and ovary in females). Except for the testicular and epididymal changes, all changes observed showed reversibility at the 6-week recovery time point. Thus, the highest non-severely toxic dose (HNSTD) of CUSP06 was 150 mg/kg via IV infusion every 3 weeks (Q3Wx2) in SD rats.

**Figure 5:**
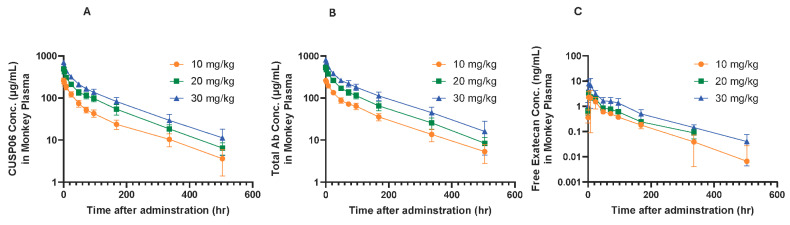
Concentration of CUSP06 (**A**), total antibody (**B**) and free exatecan (**C**) in monkey plasma after the first intravenous administration of CUSP06 at 10, 20, and 30 mg/kg from monkey toxicology study. Plasma concentrations of CUSP06 and total antibody were determined by ligand-binding assay. Plasma concentration of free exatecan was determined by LC-MS. Each value represents the mean and SD (N = 10). The concentration of free exatecan at 504 h after administration of 20 mg/kg CUSP06 is below LLOQ and is recorded as 0 ng/mL.

CUSP06 was well tolerated at all doses tested in cynomolgus monkeys. No CUSP06-related local irritation findings were found at any dose level, and no abnormal effects on central nervous system, cardiovascular and respiratory system were noted. There were no changes in plasma chemistry endpoints related to CUSP06 treatment observed in the study. The main toxicity findings included gastrointestinal toxicity manifested as transient fluid feces, reductions in reticulocytes and microscopic changes in bone marrow and thymus with a trend toward recovery after a 6-week recovery. Thus, the highest non-severely toxic dose (HNSTD) of CUSP06 was 30 mg/kg. Toxicokinetic data showed the exposure (Cmax and AUC0-504h) increased with the dose level for CUSP06 ADC, total antibody, and free exatecan payload. The low levels of free exatecan (about 1/1590-3/3470 of that of CUSP06 ADC) indicate that CUSP06 is stable in vivo in cynomolgus monkeys (Figure 5).

In summary, GLP tox study results indicate CUSP06 has a reasonable safety profile and support clinical development in CDH6-positivie human cancers.

## 4. Discussion

CDH6 expression is restricted to a few normal adult human tissues including kidney, mammary gland and thymus; however, it is overexpressed in several human malignancies including ovarian, renal carcinoma, cholangiocarcinoma, thyroid cancers and uterine serous carcinoma. CDH6 undergoes rapid internalization upon antibody binding and its membrane expression is restored upon washout of CDH6 antibody [8]. These characteristics of limited expression in normal tissues, high expression in tumor tissues, and rapid internalization upon antibody binding make CDH6 an ideal ADC target. Two CDH6-targeted ADCs have been tested in human trials. HKT288, a CDH6-targeted ADC containing a DM4 payload, was terminated due to unexpected neurologic adverse events [15]. Raludotatug Deruxtecan (R-DXd), a CDH6-targeted ADC with the Topoisomerase 1 inhibitor DXd, is currently in clinical trials for advanced ovarian and renal cancers, and exhibits promising clinical activity in ovarian cancer patients without neurologic toxicities [16]. It suggests that unexpected neurotoxicity caused by HKT288 is unlikely to be a CDH6-associated toxicity.

We report the preclinical characterization of CUSP06, a novel CDH6-targeted ADC, which supports the clinical development of CUSP06 in multiple CDH6-expressing human cancers. We used the T1000-exatecan linker payload for the construction of the ADC since (1) exatecan is a more potent topoisomerase inhibitor with better cell permeability and bystander effect compared to DXd, (2) improved solubility of the linker to balance the hydrophobicity of exatecan which allows the ease of manufacturing a DAR8 ADC, and (3) improved linker stability. CUSP06 exhibited sub-nM binding affinity to human CDH6 and its mAb showed >1000-fold selectivity over CDH9 and CDH10, two closely related cadherins. CUSP06 underwent rapid internalization in CDH6-expressing cancer cells with 50% of CUSP06 being internalized within 4 h upon treatment. Treatment of OVCAR3 cells with CUSP06, but not CUSP06 mAb or isotype ADC control, led to activation of DNA damage and cell apoptosis. CUSP06 demonstrated CDH6-dependent cytotoxicity in a panel of human ovarian cancer cell lines. In vitro cell mixing experiments demonstrated CUSP06 possessed enhanced bystander effect compared to R-DXd, which is important for anti-tumor activity in CDH6-low or heterogeneous tumors. The in vitro plasma stability data supported the excellent stability of CUSP06 over 21 days in multiple species. Together with the low-exatecan free payload level detected in the plasma from rat and monkey GLP tox studies, CUSP06 should result in lower levels of circulating free payload and reduced toxicity when it is administered to cancer patients. CUSP06 showed robust antitumor activity in multiple CDX and PDX models. In the CDH6-low kidney nephroblastoma PDX model, CUSP06 exhibited complete tumor growth inhibition (130% TGI vs. IgG-T1000-e control) whereas the R-DXd showed little antitumor activity (23% TGI vs. IgG-DXd control). Replacement of the deruxtecan linker payload of R-DXd with T1000-exatecan greatly enhanced the antitumor activity (85% TGI vs. IgG-T1000-e control), showing the clear superiority and differentiation of CUSP06 over R-DXd in this setting. Since this model does not express the drug efflux pumps Pgp and BCRP (internal data), the superior antitumor activity of CUSP06 is most likely due to the stronger bystander killing effect of T1000-e, which killed not only CDH6-high but also CDH6-low or -negative cells in this heterogeneous CDH6-expressing PDX model. CUSP06 consistently demonstrated robust antitumor activity in CDH6-high and -low expressing ovarian PDX models. In addition, CUSP06 exhibited tumor regression or robust tumor growth inhibition in CDH6-positive cholangiocarcinoma and uterine cancer PDX models. Although CDH6 expression has been reported in cholangiocarcinoma and uterine cancer, this is the first report of a CDH6-targeted ADC demonstrating efficacy in preclinical models of these two indications and highlighting the therapeutic potential of CUSP06 in these two aggressive human malignancies with unmet medical need. In summary, the preclinical pharmacology data indicate CUSP06 has promising therapeutic potential in treating CDH6-expressing malignant solid tumors including ovarian, renal, uterine cancer and cholangiocarcinoma, and in tumors that are CDH6-low, as well as CDH6-high.

CUSP06 exhibited a favorable safety profile in both species with a HNSTD of 30 mg/kg and 150 mg/kg in monkeys and rats, respectively. CUSP06 showed no lung or kidney toxicity at any doses in both rat and monkey studies. In contrast, R-DXd was reported to cause lung lesions and some kidney toxicity in the monkey study, likely due to the deruxtecan linker-payload platform [8]. The toxicity finding of CUSP06 supports the relatively safe profile of the T1000-exatecan linker payload platform [18]. The gastrointestinal toxicity (transient fluid feces) and hematological toxicities (reduction in reticulocytes and microscopic changes in bone marrow and thymus) are likely caused by the exatecan payload and are reversible. The HNSTD of 30 mg/kg in cynomolgus monkeys supports a starting dose of CUSP06 in human trials of 1.6 mg/kg (one-sixth of the human equivalent dose of HNSTD in monkey).

Collectively, the preclinical pharmacology and toxicology data indicate CUSP06 could be an effective therapeutic to treat CDH6-positive human cancers, and a phase 1 study in ovarian cancer and other advanced solid tumors is ongoing (NCT06234423).

## Figures and Tables

**Table 1:** CUSP06 Quality Attributes.

**Test Item**	**Test Results**
pH	5.5
Osmolality	310 mOsmol/kg
Protein Concentration	18.2 mg/mL
ADC Concentration	19.8 mg/mL
Purity by SEC-HPLC	97.5% Main peak/monomer
	2.4% High Molecular Weight Species (HMWS)
Drug-to-Antibody (DAR)	7.9
Bacterial Endotoxins	0.08 EU/mL
Bioburden	<1 CFU/30 mL

**Table 2:** Pharmacokinetic parameters for CUSP06 ADC and total antibody from plasma samples in PA-1 tumor-bearing mice.

Analyte	Parameter	Value 1	Value 2	Value 3	Average	SD
CUSP06 Total Antibody	T_1/2_ (h)	57.8	56.4	50.9	55.0	3.6
	T_max_ (h)	0.25	0.25	0.25	0.25	0.0
	C_max_ (µg/mL)	100.2	118.5	127.0	115.3	13.7
	AUC_0-t_ (µg/mL*h)	4964.7	7731.4	7446.3	6714.1	1521.7
CUSP06 ADC	T_1/2_ (h)	47.7	45.9	45.0	46.2	1.4
	T_max_ (h)	0.25	0.25	0.25	0.25	0.0
	C_max_ (µg/mL)	103.2	112.3	120.6	112.1	8.7
	AUC_0-t_ (µg/mL*h)	4273.2	6416.9	6258.2	5649.5	1194.5

## Data Availability

Data is contained in the article and supplemental materials. Additional inquiries can be directed to the corresponding authors.

## Supplementary Materials

The following supporting information can be downloaded at: https://www.mdpi.com/article/10.3390/pharmaceutics17081049/s1, Figure S1: Reverse phase HPLC spectrum of fully conjugated CUSP06. (A) Entire RP-HPLC spectrum of CUSP06. LC0-LC1 stands for light chain with 0 or 1 T1000-exatecan. HC0-3 stands for heavy chain with 0-3 T1000-exatecan. The table of DAR calculation shows CUSP06 possesses the DAR value of 7.9 with the purity at 97%. (B) Identification and assignment of each peak from RP-HPLC by mass spectroscopy; Figure S2: Characterization of two IgG-T1000-e controls. (A) Tabulated summary of the purity and DAR value for the two IgG-T1000-e controls. (B) The purity of the two IgG-T1000-e controls by Size Exclusion Chromatography; Figure S3: Characterization of R-DXd. (A) Tabulated summary of the purity and DAR value for R-DXd. (B) The purity of R-DXd is determined by Size Exclusion Chromatography; Figure S4: Measurement of Pgp and BCRP proteins in nephroblastoma LD-2511 PDX tumor by IHC.
