# Supplementary material for: CUSP06, a Novel CDH6-Targeted Antibody-Drug Conjugate, Demonstrates Antitumor Efficacy in Multiple CDH6-Expressing Human Cancer Models
Source: Pharmaceutics. 2025 Aug 13;17(8):1049. doi: 10.3390/pharmaceutics17081049 (PMC12388900; doi:10.3390/pharmaceutics17081049)
Supplement: Supplementary file 1 [file pharmaceutics-17-01049-s001.zip › pharmaceutics-3672287-supplementary.pdf]

Supplementary Figure S1

1A: Reverse Phase HPLC spectrum of Fully conjugated CUSP06

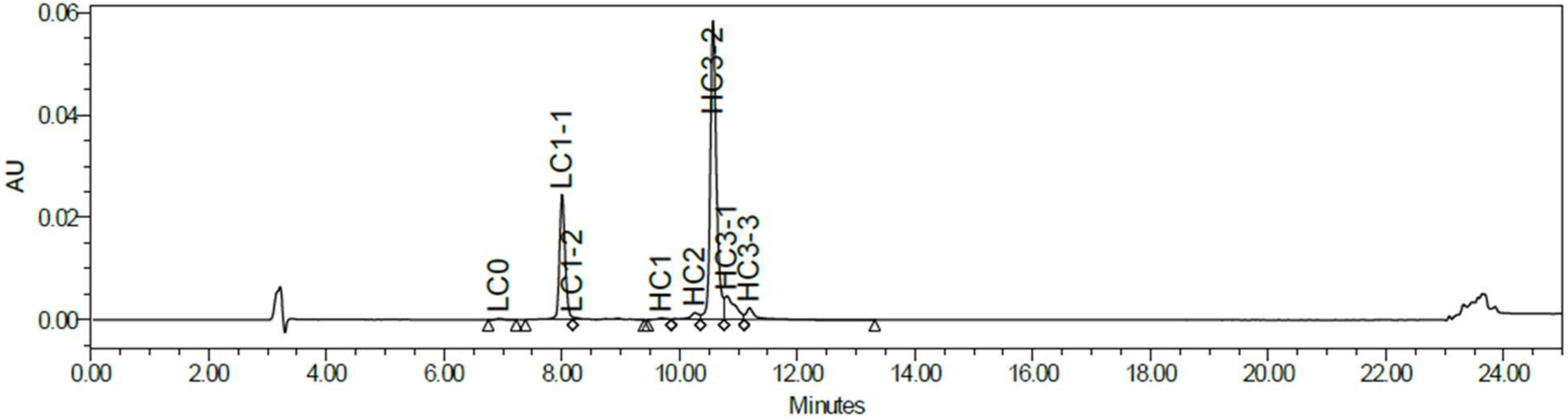

Peak	Area	% Area	Contribution to DAR	DAR
LC0	0.2582	0.0105	0	0
LC1	24.3453	0.9895	2	1.98
			**Total LC DAR**	**1.98**
HC0	0	0.0000	0	0.00
HC1	0.4866	0.0065	2	0.01
HC2	2.1693	0.0288	4	0.12
HC3	72.7406	0.9648	6	5.79
			**Total HC DAR**	**5.92**
			**Total DAR (HC + LC)**	**7.90**

$$\text{Average DAR} = \left(\frac{LC_1}{(LC_0 + LC_1)} \times 2\right) + \left(\frac{HC_1}{(HC_0 + HC_1 + HC_2 + HC_3)} \times 2\right) + \left(\frac{HC_2}{(HC_0 + HC_1 + HC_2 + HC_3)} \times 4\right) + \left(\frac{HC_3}{(HC_0 + HC_1 + HC_2 + HC_3)} \times 6\right)$$

Supplementary Figure S1

1B: Peak identification and assignment via Mass Spectroscopy

Zoom in

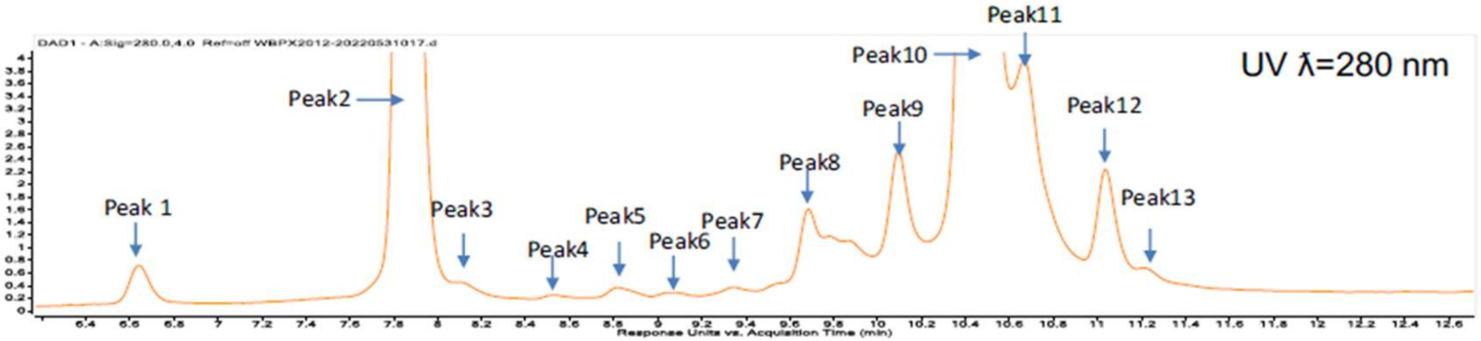

Peak Name	Retention Time	Theoretical Mass (Da)[1]	Measured Mass (Da)	Peak Assigned
Peak1	6.64	23406.1	23406.7	LC0 (fully)
Peak2	7.86	25151.9	25151.0	LC1(fully)
Peak3	8.10	25151.9	25151.0	LC1 related(fully)
Peak4	8.53	25151.9	25107.0	LC1 related(-45 Da, fully)
Peak5	8.82	25151.9	25153.7	LC1 related(fully)
Peak6	9.07	25151.9	25153.7	LC1 related(fully)
Peak7	9.35	50813.7	50816.3	HC0(G0F, fully)
Peak8	9.69	52557.5	52560.1	HC1(G0F, fully)
Peak9	10.10	54298.7	54301.8	HC2(G0F, fully)
Peak10	10.44	56048.5	56048.9	HC3 (G0F, fully)
Peak11	10.67	54603.2	54603.3	HC3 (Deglycosylated, fully)
Peak12	11.04	56048.5	56050.9	HC3 related(G0F, fully)
Peak13	11.21	56048.5	56050.9	HC3 related(G0F, fully)

Where:
- LC0 = unconjugated Light Chain
- LC1 = Light chain with one LP
- HC0 = unconjugated Heavy Chain
- HC1 = Heavy chain with one LP
- HC2 = Heavy chain with two LP
- HC3 = Heavy chain with three LP

And
- Mass LC = 23406.1 Da
- Mass HC =50813.7 Da
- Mass LP = 1745 Da

Supplemental Figure S2: Characterization of IgG-ADC controls

A

	Purity (%) [1]	Drug Antibody Ratio
Bezlotoxumab-T1000-e	97.3	7.37
Rituximab-T1000-e	96.9	7.65

1. The purity of IgG-T1000-e control was determined by size exclusion column.

B

Characterization of Bezlotoxumab-T1000-e purity by SEC

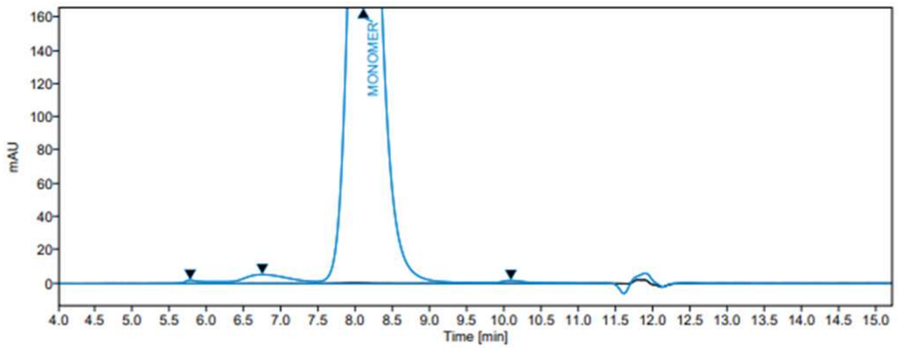

Characterization of Rituximab-T1000-e purity by SEC

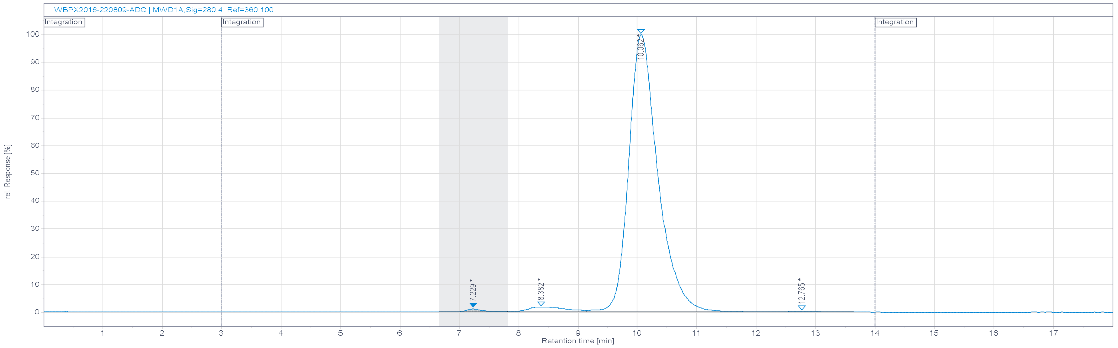

Supplemental Figure S3: Characterization of R-DXd

A

	Purity (%) [1]	Drug Antibody Ratio
R-DXd	97.3	7.7

1. The purity of R-DXd was determined by size exclusion column

B

Characterization of R-DXd by SEC：

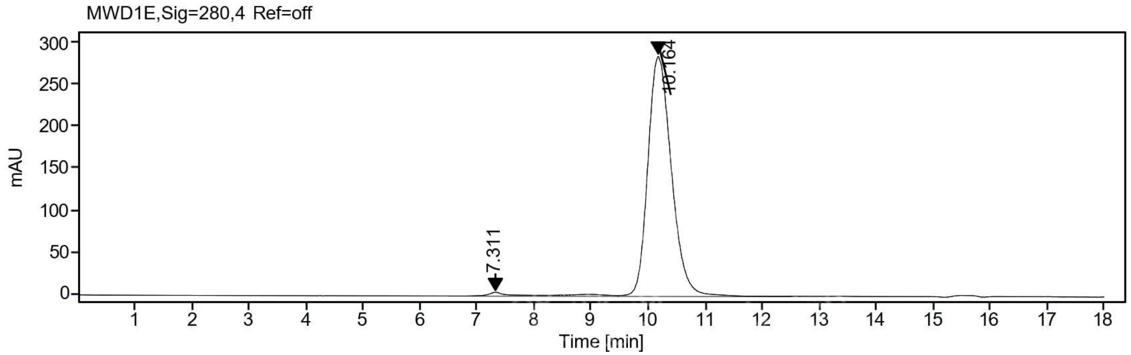

Signal: MWD1E,Sig=280,4 Ref=off

Name	Retention Time	Area	Area%	Peak Signal To Noise
	7.31	223.3211	2.66	
	10.16	8179.5167	97.34	
	Sum	8402.8378		

Supplemental Figure S4: Measurement of Pgp and BCRP protein in LD-2511 PDX tumor samples by IHC

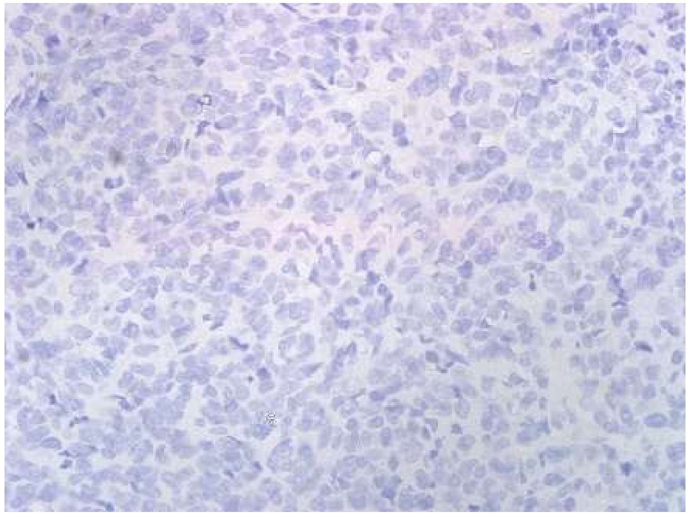

BCRP

400X

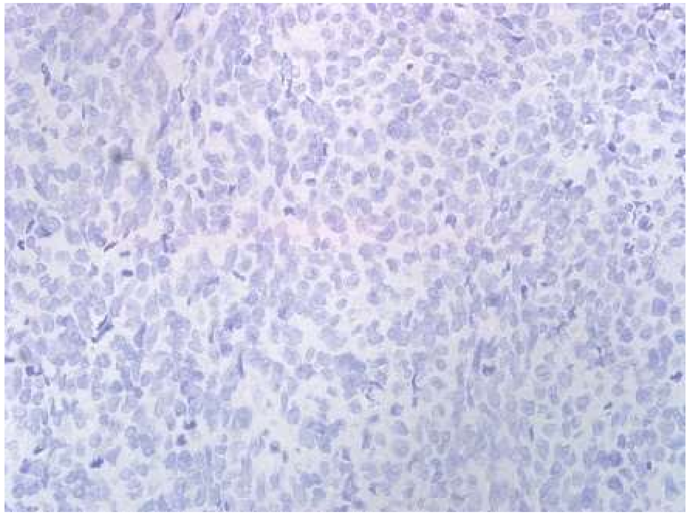

Pgp

400X
